# Effects of COVID-19 Pandemic on Reported Lyme Disease, United States, 2020

**DOI:** 10.3201/eid2710.210903

**Published:** 2021-10

**Authors:** David W. McCormick, Kiersten J. Kugeler, Grace E. Marx, Praveena Jayanthi, Stephanie Dietz, Paul Mead, Alison F. Hinckley

**Affiliations:** Centers for Disease Control and Prevention, Fort Collins, Colorado, USA. (D.W. McCormick, K.J. Kugeler, G.E. Marx, P. Mead, A.F. Hinckley);; ICF International Inc., Atlanta, Georgia, USA (P. Jayanthi);; Centers for Disease Control and Prevention, Atlanta (P. Jayanthi, S. Dietz)

**Keywords:** Lyme disease, tickborne diseases, vector-borne infections, surveillance, healthcare-seeking behavior, respiratory infections, severe acute respiratory syndrome coronavirus 2, SARS-CoV-2, SARS, COVID-19, coronavirus disease, zoonoses, viruses, coronavirus

## Abstract

Surveys indicate US residents spent more time outdoors in 2020 than in 2019, but fewer tick bite–related emergency department visits and Lyme disease laboratory tests were reported. Despite ongoing exposure, Lyme disease case reporting for 2020 might be artificially reduced due to coronavirus disease–associated changes in healthcare-seeking behavior.

The coronavirus disease (COVID-19) pandemic has altered how humans interact with their environment and the healthcare system (*1*,*2*), and strained resources have limited the ability of state and local health departments to respond to reports of notifiable diseases (*3*). The Centers for Disease Control and Prevention (CDC) typically is notified of 30,000–40,000 Lyme disease cases annually (*4*), but the COVID-19 pandemic likely will affect the case counts. Most Lyme disease cases are acquired in spring and early summer (*5*); in 2020, these seasons coincided with the initial spread of COVID-19 and widespread stay-at-home orders. We explored 4 data sources to assess how the COVID-19 pandemic might have influenced tick bite risk and associated healthcare-seeking practices and affected reported Lyme disease cases for 2020. 

The pathway for Lyme disease case reporting begins with environmental risk and culminates with case notification to CDC (Appendix Figure). Environmental risk is relatively stable in high-incidence areas and driven by ecologic factors unaffected by COVID-19 (*6*). The pandemic might have altered the frequency of outdoor activities and probability of encountering ticks, healthcare-seeking and provider services patterns, and case investigation and reporting. The data sources we used measure changes in time spent outdoors, information-seeking patterns for tick removal, emergency department (ED) visits for tick bites, and laboratory testing for Lyme disease. This analysis was considered nonhuman subjects research by CDC.

To assess potential behavior shifts that might have increased risk for tick encounters, we analyzed data from Porter Novelli’s PN View 360+ consumer survey (*7*). Among 4,013 participants who responded to the survey distributed during July 31–August 9, 2020, approximately half (49.9%) reported that they had spent a lot more time or slightly more time outdoors by that point in 2020 compared with prior years. Only 20.9% of respondents reported spending less time outdoors in 2020.

To indirectly assess frequency of tick encounters in 2020 compared with prior years, we evaluated total monthly visits during 2018–2020 to a CDC website describing tick removal (*8*). Visits to this website typically increase during late spring and summer and again in October, when most bites from blacklegged ticks (*Ixodes scapularis* and *Ixodes pacificus*) occur (*5*). We observed 818,167 website visits during 2020, ≈25% more than in 2019 (681,021) and 2018 (630,839) (Figure).

**Figure:**
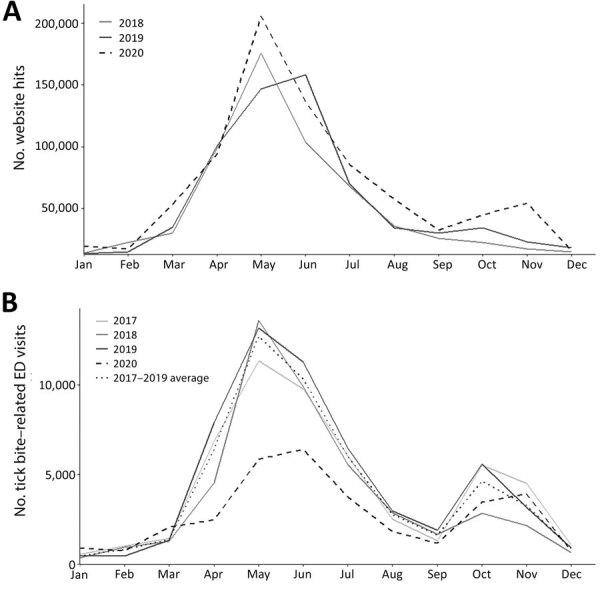
Comparison of visits to the Centers for Disease Control and Prevention (CDC) website on tick removal, 2018–2020, and to the ED for tick-bite related chief complaints, 2017–2020, United States. A) Website visits per month for https://www.cdc.gov/ticks/removing_a_tick.html. B) ED visits by month in which the chief health complaint was tick bite. Comparison of 2020 to the average of the previous 4 years is shown. ED, emergency department.

To assess patterns related to healthcare-seeking for tick encounters, we identified ED visits for tick bites by using the National Syndromic Surveillance Program (NSSP) BioSense platform (*9*). ED visits for tick bites decreased in 2020 from 2019 in both total number and rate per 100,000 ED visits (Figure). The largest relative decreases were observed in May. During 2017–2019, the average number of ED visits for tick bites during the month of May was 12,693, an average rate of 145/100,000 ED visits. During May 2020, only 5,845 ED visits for tick bites occurred, a rate of 89/100,000 ED visits.

We quantified cumulative counts and percent positivity of serologic tests for Lyme disease performed by an independent clinical laboratory. Lyme disease testing volume decreased from 2019 to 2020; 25.0% fewer tests were performed, and test positivity decreased slightly to <1% (Table).

**Table:** Number and percent positive for Lyme disease tests performed by a large commercial laboratory and percent decrease in 2020 compared with 2019, United States*

Testing tier	2019			2020		% Decrease in testing volume (95% CI)	Absolute difference in % positive (95% CI)
	Total tests	% Positive		Total tests	% Positive		
First tier†	925,939	9.6		691,453	9.2	25.3 (25.2–25.4)	0.3 (0.2–0.4)
Second tier‡	422,801	11.0		320,616	10.2	24.2 (24.1–24.3)	0.8 (0.6–0.9)
Total	1,348,740	10.0		1,012,069	9.5	25.0 (25.1–24.9)	0.5 (0.4–0.6)

*Percent positive indicates the percentage of the total laboratory tests that were positive for each test tier and overall. The percent decrease in testing volume shows the percentage decrease in total tests performed by tier and overall for 2020 compared with 2019. Two-tier testing for Lyme disease is recommended, whereby specimens positive or equivocal on the first tier are subjected to the second tier. Additional details about testing tiers are available at https://www.cdc.gov/lyme/diagnosistesting/index.html.

†First-tier tests include enzyme immunoassays for IgM/IgG combined, IgM alone, and C6 antigen.

‡Second-tier tests include immunoblot for IgM or IgG.

During the first wave of the COVID-19 pandemic in 2020, the US population spent more time outdoors and visited a CDC website describing safe tick removal more frequently than during prior years. However, fewer persons sought care for tick bites, and substantially fewer laboratory tests for Lyme disease were ordered. These findings suggest that the risk of acquiring Lyme disease was similar or potentially higher in 2020 compared with risk during prior years, but fewer persons sought care, and fewer positive laboratory reports were referred for case investigation. Consequently, we anticipate that, once ultimately finalized, the official number of confirmed and probable Lyme disease cases in 2020 will be substantially lower than that for prior years.

One limitation of our study is that data sources we examined represent national trends and are indirect surrogates for Lyme disease risk and reporting, which vary geographically. Visits to a website describing tick removal might not correspond with finding an attached tick. Available data on laboratory testing represents 1 independent clinical laboratory; other commercial or academic laboratories might not have experienced a similar decrease in testing. Data sources associated with telehealth utilization and prescription claims could provide additional insights into the diagnosis and treatment for Lyme disease in 2020.

Already an issue in high-incidence states, the pandemic has highlighted the need for alternative Lyme disease surveillance strategies that rely less on human resources. An anticipated and potentially substantial decrease in reported Lyme disease in 2020 likely reflects the effects of the COVID-19 pandemic rather than a true change in Lyme disease incidence. Decreased reporting also could render 2020 inconsistent with long-term trends and changes in the epidemiology of the disease. Although nonpharmaceutical interventions for COVID-19 have mitigated the transmission of respiratory pathogens (*10*), these results suggest the behavioral and reporting changes seen for Lyme disease might extend to other nonrespiratory diseases.

Appendix Additional information on effects of COVID-19 pandemic on reported Lyme disease, United States, 2020.

## References

[1] Hartnett KP, Kite-Powell A, DeVies J, Coletta MA, Boehmer TK, Adjemian J, et al.; National Syndromic Surveillance Program Community of Practice. Impact of the COVID-19 pandemic on emergency department visits—United States, January 1, 2019–May 30, 2020. MMWR Morb Mortal Wkly Rep. 2020;69:699–704. 10.15585/mmwr.mm6923e1 32525856 PMC7315789

[2] Czeisler MÉ, Marynak K, Clarke KEN, Salah Z, Shakya I, Thierry JM, et al. Delay or avoidance of medical care because of COVID-19–related concerns—United States, June 2020. MMWR Morb Mortal Wkly Rep. 2020;69:1250–7. 10.15585/mmwr.mm6936a4 32915166 PMC7499838

[3] Weber L, Ungar L, Smith MR, Recht H, Barry-Jester AM. Hollowed out public health system faces more cuts amid virus. Associated Press. 2020 Jul 1 [cited 2021 Apr 20]. https://apnews.com/article/b4c4bb2731da9611e6da5b6f9a52717a

[4] Schwartz AM, Hinckley AF, Mead PS, Hook SA, Kugeler KJ. Surveillance for Lyme disease—United States, 2008–2015. MMWR Surveill Summ. 2017;66:1–12. 10.15585/mmwr.ss6622a1 29120995 PMC5829628

[5] Mead PS. Epidemiology of Lyme disease. Infect Dis Clin North Am. 2015;29:187–210. 10.1016/j.idc.2015.02.010 25999219

[6] Burtis JC, Sullivan P, Levi T, Oggenfuss K, Fahey TJ, Ostfeld RS. The impact of temperature and precipitation on blacklegged tick activity and Lyme disease incidence in endemic and emerging regions. Parasit Vectors. 2016;9:606. 10.1186/s13071-016-1894-6 27887625 PMC5124264

[7] Porter Novelli. ConsumerStyles & YouthStyles 2021 [cited 2021 Apr 20]. http://styles.porternovelli.com/consumer-youthstyles

[8] Centers for Disease Control and Prevention. Tick removal [cited 2021 Apr 20]. https://www.cdc.gov/ticks/removing_a_tick.html

[9] Marx GE, Spillane M, Beck A, Stein Z, Powell AK, Hinckley AF. Emergency department visits for tick bites—United States, January 2017–December 2019. MMWR Morb Mortal Wkly Rep. 2021;70:612–6. 10.15585/mmwr.mm7017a2 33914718 PMC8084121

[10] Olsen SJ, Azziz-Baumgartner E, Budd AP, Brammer L, Sullivan S, Pineda RF, et al. Decreased influenza activity during the COVID-19 pandemic-United States, Australia, Chile, and South Africa, 2020. Am J Transplant. 2020;20:3681–5. 10.1111/ajt.16381 33264506 PMC7753605

