# Supplementary material for: Effects of COVID-19 Pandemic on Reported Lyme Disease, United States, 2020
Source: Emerg Infect Dis. 2021 Oct;27(10):2715–7. doi: 10.3201/eid2710.210903 (PMC8462321; doi:10.3201/eid2710.210903)
Supplement: Appendix — Additional information on effects of COVID-19 pandemic on reported Lyme disease, United States, 2020. [file 21-0903-Techapp-s1.pdf]

Article DOI: https://doi.org/10.3201/eid2710.210903

Effects of COVID-19 Pandemic on Reported Lyme Disease, United States, 2020

Appendix

Additional Methods

We used 4 data sources to evaluate the effects of the coronavirus disease (COVID-19) pandemic on Lyme disease reporting: Porter Novelli's PN View 360+ consumer survey (https://styles.porternovelli.com/consumer-youthstyles); the National Syndromic Surveillance Program (NSSP) BioSense platform (https://www.cdc.gov/nssp/index.html); data on Lyme disease testing performed at a large commercial laboratory; and visits to a the Centers for Disease Control and Prevention website describing safe tick removal (https://www.cdc.gov/ticks/removing_a_tick.html). We provide methods for analysis of website visits in the manuscript.

We evaluated potential exposure to ticks in 2020 compared with prior years by examining responses to a question from the Porter Novelli's PN View 360+ consumer survey asking participants about the amount of time spent outdoors. This survey is a nationwide cross-sectional survey administered online to adults ≥18 years of age. Data are weighted by age, sex, region, race, and education to match current population estimates for the United States.

We evaluated tick bite-related visits to emergency departments (EDs) using the National Syndromic Surveillance Program (NSSP) BioSense platform. NSSP includes data from ≈70% of nonfederal EDs in the United States.

To determine trends in laboratory testing for Lyme disease, we analyzed data on serologic tests for Lyme disease performed by an independent clinical laboratory (Lab A) during February–December in 2019 and in 2020. Consistent reporting to this platform began in February 2019, so we excluded data from January 2020 to ensure comparability. We identified

tests by using Logical Observation Identifiers Names and Codes, LOINC version 2.70 (https://loinc.org), and data were sent from Lab A to NSSP every 10 minutes via HL7 messages.

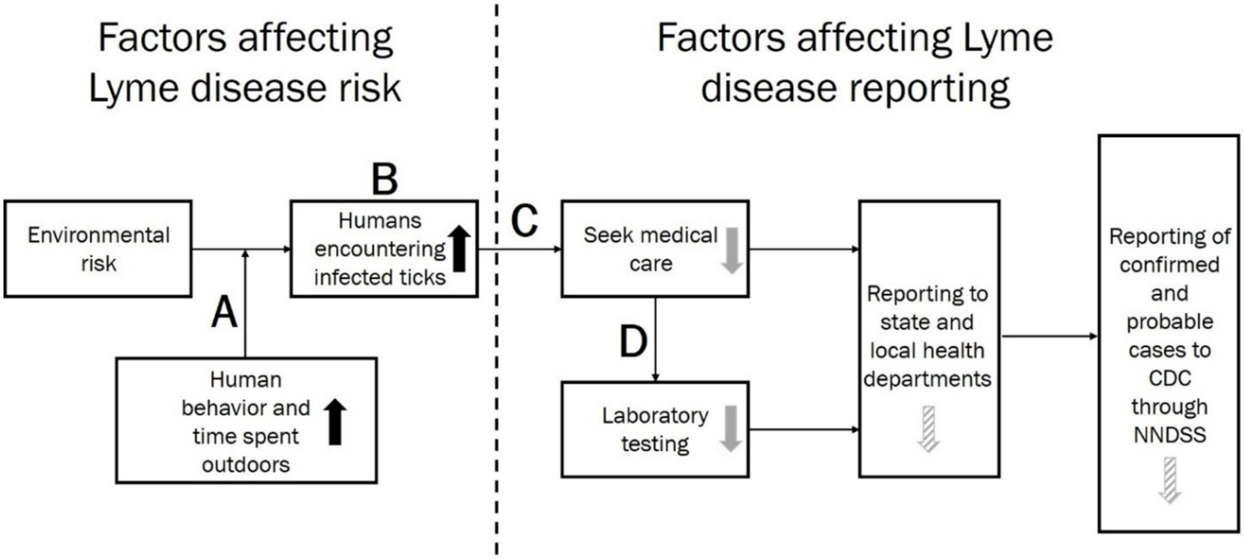

Appendix Figure. Factors affecting Lyme disease risk and reporting during the coronavirus disease (COVID-19) pandemic, United States, 2020. The left side shows pathways for encountering infected ticks; the right side shows pathways for seeking medical care and reporting of possible Lyme disease cases to state and local health departments for investigation and reporting cases that meet the confirmed and probable case definition to the Centers for Disease Control and Prevention (CDC). Black arrows indicate increased activity due to the COVID-19 pandemic. Solid gray arrows indicate decreased activity due to the COVID-19 pandemic. Dashed gray arrows indicate predicted decreases for data are not currently available. Letters indicate data sources: A) Porter Novelli ConsumerStyles survey (https://styles.porternovelli.com/consumer-youthstyles); B) visits to Centers for Disease Control and Prevention website describing safe tick removal (https://www.cdc.gov/ticks/removing_a_tick.html); C) the National Syndromic Surveillance Program (NSSP) BioSense platform (https://www.cdc.gov/nssp/index.html); D) reported Lyme disease testing performed by a large commercial laboratory. NNDSS, National Notifiable Diseases Surveillance System.
